# Genes but Not Genomes Reveal Bacterial Domestication of *Lactococcus Lactis*

**DOI:** 10.1371/journal.pone.0015306

**Published:** 2010-12-17

**Authors:** Delphine Passerini, Charlotte Beltramo, Michele Coddeville, Yves Quentin, Paul Ritzenthaler, Marie-Line Daveran-Mingot, Pascal Le Bourgeois

**Affiliations:** 1 Université de Toulouse, Université Paul Sabatier, Toulouse, France; 2 Laboratoire de Microbiologie et de Génétique Moléculaires, CNRS, Toulouse, France; 3 SOREDAB SAS, La Tremblaye, La Boissière-Ecole, France; University of Hyderabad, India

## Abstract

**Background:**

The population structure and diversity of *Lactococcus lactis* subsp. *lactis*, a major industrial bacterium involved in milk fermentation, was determined at both gene and genome level. Seventy-six lactococcal isolates of various origins were studied by different genotyping methods and thirty-six strains displaying unique macrorestriction fingerprints were analyzed by a new multilocus sequence typing (MLST) scheme. This gene-based analysis was compared to genomic characteristics determined by pulsed-field gel electrophoresis (PFGE).

**Methodology/Principal Findings:**

The MLST analysis revealed that *L. lactis* subsp. *lactis* is essentially clonal with infrequent intra- and intergenic recombination; also, despite its taxonomical classification as a subspecies, it displays a genetic diversity as substantial as that within several other bacterial species. Genome-based analysis revealed a genome size variability of 20%, a value typical of bacteria inhabiting different ecological niches, and that suggests a large pan-genome for this subspecies. However, the genomic characteristics (macrorestriction pattern, genome or chromosome size, plasmid content) did not correlate to the MLST-based phylogeny, with strains from the same sequence type (ST) differing by up to 230 kb in genome size.

**Conclusion/Significance:**

The gene-based phylogeny was not fully consistent with the traditional classification into dairy and non-dairy strains but supported a new classification based on ecological separation between “environmental” strains, the main contributors to the genetic diversity within the subspecies, and “domesticated” strains, subject to recent genetic bottlenecks. Comparison between gene- and genome-based analyses revealed little relationship between core and dispensable genome phylogenies, indicating that clonal diversification and phenotypic variability of the “domesticated” strains essentially arose through substantial genomic flux within the dispensable genome.

## Introduction

The massively increasing amount of genomic data becoming available is raising questions about the classical view of bacterial species, particularly in terms of gene content. Beginning with the pioneering observation of Lan & Reeves [1], it is now established that sequencing a single genome fails to describe the genetic content of the species, and intraspecies variation needs to be considered to gain insight into the full “species genome”. This genome, alternatively named the pan-genome, is composed of a core genome made up of genes ubiquitously present in all strains of a given species, and a dispensable genome containing genes found only in single strains or particular lineages. Depending on the species and the number of strains sequenced, the core genome only represents from 40% to 80% of a single genome, and the pan-genome may be almost 4 times the size of a genome in a single strain [2]–[5]. Understanding the extent of the genetic diversity within a species should help the choice of strains to be sequenced for pan-genome characterization. A powerful method for population genetic studies is multilocus sequence typing (MLST) [6], a method based on the sequencing of a limited number (generally five to seven) genes of the core genome. MLST outperforms restriction- or other PCR-based typing methods, because it provides information about key features of the evolutionary history, the population structure, and long-term epidemiology of bacterial species [7], [8]. Although MLST has been principally used to study the major bacterial pathogens, several recent MLST schemes have been developed for lactic acid bacteria (LAB), the most important group of microorganisms used for food processing, including the species *Lactobacillus plantarum* [9], *Lactobacillus casei* [10], [11], *Oenococcus oeni* [12], and *Streptococcus thermophilus* [13].

*Lactococcus lactis* is the major LAB species used in milk fermentation, a preservation process probably first developed in the Early Neolithic [14]. This type of fermentation involving natural starters has been used empirically at a small scale for thousands of years, through the practice of back-slopping. Industrial scale fermentation started in the early-20^th^ century with the use of defined single- and multiple-strain commercial starters [15]. *L. lactis* is a microorganism that is generally recognized as safe (GRAS), and is also now used as a cell factory for production of recombinant proteins [16], and as a therapeutic drug delivery vector [17], [18]. Taxonomically, it is a mesophilic Gram-positive species related to the *Streptococcaceae* [19]; it is subdivided into three subspecies, *L. lactis* subsp. *hordniae*, *L. lactis* subsp. *lactis* (including the biovar *diacetylactis*), and *L. lactis* subsp. *cremoris*. The two latter subspecies differ by less than 0.7% in their 16S rDNA sequences [20] but display an average of only 85% DNA identity at the genome level [21], a value slightly higher than that between *Escherichia coli* and *Salmonella typhimurium* [22]. *L. lactis* subsp. *lactis* is found in various environments including animal sources, dairy products and plant surfaces [23], [24], whereas the subspecies *cremoris* is only isolated from raw milk and dairy products [24], [25], with few exceptions [26], [27]. This ability of *L. lactis* subsp. *lactis* to colonize a larger ecological niche is associated to a greater genomic diversity, as revealed by DNA-fingerprinting analyses including random amplification of polymorphic DNA (RAPD) and pulsed-field gel electrophoresis (PFGE): subspecies *lactis* strains are spread across many clusters whereas subspecies *cremoris* strains are grouped in a small number of closely related clusters [24], [28], [29]. Dairy strains of both subspecies tend to display lower diversity than non-dairy strains [26], [30], [31]. The sole MLST scheme reported to date for *L. lactis* analyzed the nucleotide variability at five genetic loci of 89 *L. lactis* subsp. *lactis* and *L. lactis* subsp. *cremoris* isolates [31] and substantiated these previous observations. However, this study gave little information about the population structure and gene diversity/evolution of the species.

We report an analysis of the diversity of *L. lactis* subsp. *lactis* strains at both gene (MLST) and genome (PFGE) levels. Seventy-six lactococcal isolates were analyzed by various molecular typing methods to validate a collection of 36 strains. A new MLST scheme was constructed using a rational “top-down” approach, and the population structure, the genetic diversity, and a gene evolution model were estimated for this subspecies. Genome characteristics, including chromosome size and plasmid content, and genomic relatedness were estimated by PFGE analysis. The findings are informative about the origin and the ecology of the strains analyzed.

## Results

### Methodological considerations about bacterial genetic diversity

MLST is currently considered to be the gold standard method for studying strain relationships and the population structure of bacteria [7]. Though dozens of MLST schemes have been developed to date, many of them do not follow the good practices for the rational development of MLST schemes [32], such as appropriate initial population sampling, rational choice of the genetic loci to be characterized, and use of suitable statistics (referred in this study as the quantity calculated from a set of data) for a robust estimation of genetic diversity.

### Methodological considerations: 1) Constitution of the *L. lactis* subsp. *lactis* sample collection

Exploration of bacterial diversity by MLST requires the use of an appropriate strain collection validated by different genotyping methods. We therefore obtained 76 isolates from several public and industrial collections and used various phenotypic (ability to grow in milk or to utilize lactose) and genotypic (ribotyping, ARDRA, and partial 16S rDNA sequencing) methods to study them. Eighteen strains were discarded from the analysis because they presented ARDRA and/or ribotyping results that were either ambiguous or inconsistent with an affiliation to the *L. lactis* subsp. *lactis* group. Indeed, partial 16S rDNA sequencing (data not shown) revealed that these strains belonged either to the *cremoris* subspecies or to genera other than *Lactococcus* (*Enterococcus faecalis*, *Enterococcus pseudoavium, Lactobacillus casei*, and *Leuconostoc citreum*). The remaining 57 strains were subjected to *Sma*I-macrorestriction analysis by PFGE; an additional 21 strains were thereby excluded because they displayed a macrorestriction fingerprint identical to that of strains already selected for the collection. This observation enlightens strain redundancy that may exist in laboratory collections, probably because these collections are mostly constituted from phenotypic characterizations. Moreover, the PFGE analysis not only confirmed the close genomic relationships between known pairs of strains (IL594 and its plasmid-free derivative IL1403 [33], S86 and its [Lac]^-^ spontaneous derivative S86-B), but also identified unexpected close relatedness between some other pairs of strains, such as LD01/LD02 and UCMA5713/UCMA5733. As strains of each pair differed by only one *Sma*I fragment identified as the lactose plasmid (by Southern-hybridization against the *lacE* gene carried on the lactose plasmid [34]), the pairs presumably correspond to [Lac] variants of the same strains. Thus, the bacterial collection validated for this study consisted of 36 strains displaying different *Sma*I macrorestriction patterns (pulsotype). Twenty-three of the strains originated from dairy environments, such as milk, fermented products, or starter strains, and 13 strains had been isolated from various non-dairy environments, including plants, animal skin, and sourdough bread (Table S1, provided as supplementary material).

### Methodological considerations: 2) Rational development of a MLST scheme for lactococcal population study

Some of the criteria for the choice of the gene set have changed since the first proposals of MLST schemes. For instance, targeting only housekeeping genes emerged as optional [35], whereas choosing loci that follow the same evolutionary route (i.e. displaying congruent tree topology) may be essential to minimize noise when extracting phylogenetic signals from concatenated sequences [36]. According to good practice for the rational development of a MLST scheme [32], we developed a new lactococcal MLST scheme using a four-step “top-down” approach. First, 33 loci were evaluated by *in silico* analysis using publicly available lactococcal nucleotide sequences, with emphasis on DNA polymorphism, chromosomal distribution, and gene paralogy. These loci were either markers commonly used in other eubacterial MLST schemes, including the five loci used for the previous *L. lactis* MLST scheme (*atpA*, *bcaT*, *pepN, pepXP*, and *rpoA*) [31], or indicators of the overall rate of genome divergence between bacterial species (*recN*, *glyA*, and *metS*[*metG*]) [37].

Fourteen loci (*bcaT*, *glyA*, *pgk*, *dprA*, *pfk*, *comX*, *metS*, *mutX*, *rpoA*, *recN*, *tkt*, *pepXP*, *pdp*, and *xerS*) fulfilling the above criteria were selected for the second step of the analysis: the determination of the entire DNA sequences of these genes in a subset of 13 strains of the collection displaying various levels of genomic diversity as assessed by PFGE analysis (data not shown). Five loci were rejected following this analysis: three loci (*comX*, *mutX*, and *xerS*) gave sequence data of too short length or of poor quality, one (*rpoA*) provided only a weak phylogenetic signal with only three SNPs among the 13 strains, and one (*metS*[*metG*]) did not clearly separate the *lactis* and *cremoris* subspecies. The findings for the *metS*[*metG*] marker support the recent observation that some genes encoding aminoacid-tRNA synthetases, though belonging to the core of the minimum bacterial gene set [38], [39], may be horizontally transferred between species or subspecies [31], [40], [41].

The third step of the MLST scheme design consisted of selecting the most polymorphic region of ≈500 bp in length for each of the nine loci selected, and determining the sequence of this region in each strain of the entire validated collection, excluding one strain from each parent/derivative pair (Table S1). For each locus, the quality of the phylogenetic signal was investigated by split decomposition analysis [42], a method allowing the visualization of conflicting signals in phylogenetic studies by representing incompatibilities between data as networks. Five loci (*pepXP*, *recN*, *pdp*, *pgk*, and *glyA*) gave classical tree-like structures, whereas three loci (*dprA*, *pfk*, and *bcaT*) gave little network-like structures (Fig. S1, provided as Supporting Information). In contrast, the *tkt* locus displayed a split-network arrangement typical of phylogenetic incompatibilities within data (Fig. S1) and was rejected from the scheme. Comparative analysis with four different lactococcal genomes revealed that *tkt* flanked a genomic island (data not shown), a chromosomal position prone to intragenic recombination leading to phylogenetic incongruence among *Escherichia coli* strains [5].

Finally, the MLST scheme was optimized to give the best compromise between a small number of loci to sequence and a large number of sequence types (STs) generated. With 10 to 13 alleles per locus, the combination of the eight loci selected allowed 26 STs to be distinguished among the 32 strains analyzed. As the five loci displaying no conflicting trees were uniformly distributed on the three sequenced genomes (data not shown), they were used as the backbone for the MLST scheme. This five-locus scheme generated 23 STs and addition of *dprA*, *pfk*, or *bcaT* loci individually generated 24, 25, and 25 STs respectively, whereas only the simultaneous addition of *pfk* and *bcaT* increased the number of STs to 26. These observations allowed the rejection of the *dprA* locus from the scheme. In addition, the *pfk* and *bcaT* loci are located only 51 kbp apart in the three sequenced genomes, and the phylogenetic tree generated by the concatenated sequence from the six-locus scheme did not change either its topology or its robustness relative to the seven-loci tree (data not shown); consequently, the *pfk* locus was removed from the scheme.

In conclusion, the new MLST scheme targeted six loci uniformly distributed along the chromosome (Fig. S2, provided as Supporting Information) and displaying little phylogenetic inconsistency: three housekeeping genes (*glyA*, *pgk*, and *pdp*), two catabolic genes (*bcaT*, and *pepXP*) genes, and one gene of the SOS regulon (*recN*). Note that two of these loci (*recN* and *glyA*) belong to the gene set identified as the best predictors of whole-genomes relatedness [37], whereas only two loci described in the previous lactococcal MLST scheme [31], *bcaT* and *pepXP*, were retained. The new MLST scheme allowed 25 ST to be distinguished among the 32 strains analyzed.

### Methodological considerations: 3) choice of appropriate statistics for genetic diversity estimation

Survey of MLST studies showed that several statistics are used, sometimes with redundancy, to estimate the bacterial genetic diversity. In addition, many studies compare the level of genetic diversity between bacterial species although the loci selected for each MLST scheme are generally different and may have diverse evolutionary rates. Therefore, selecting which statistic to use for estimation of bacterial gene diversity level is not a trivial task since no comparative study has been performed to date. We analyzed the robustness of two statistics most commonly used in MLST studies -the percentage of variable sites and the nucleotide diversity (π, [43])- using concatenated DNA sequences obtained from MLST data of several bacterial species (Table 1). The maximal nucleotide diversity (π_MAX_), defined as the number of nucleotide differences per site between the two most divergent sequences within the population, was also included (Table 1). The sensitivity of these statistics to the sample size was estimated by calculating their values both from all available STs and then from a random sample of 25 STs (the size of our ST sample). As isolate redundancy within each ST cannot be exclude in absence of complementary genotypic characterization, this analysis was performed using only one sample from each ST (non redundant STs).

**Table 1:** Study of some statistics used to measure gene diversity according to the size and the population structure of strain samples.

		% of variable sites		π b (%)		π_MAX_ (%)		π_MAX_/π ratio
Species	n a	(Pop.)	(25 STs)	(Pop.)	(25 STs)	(Pop.)	(25 STs)	(Pop.)
*S. pneumoniae*	3913	44.38	6.43	1.07	1.15±0.14	16.21 (5.05) c	3.09	15.15 (4.71) c
*S. aureus*	1557	30.98	4.75	0.78	0.89±0.06	17.67 (3.50) d	1.75	22.65 (4.49) d
*H. pylori*	1120	49.91	15.67	4.11±0.02	3.88±0.12	8.11	5.70	1.97
*H. influenzae*	672	19.33	9.68	2.40±0.04	2.46±0.21	5.50	4.58	2.29
*B. cereus*	544	26.29	13.68	4.40±0.07	4.26±0.17	10.53	6.08	2.39
*E. faecium*	521	19.46	14.86	1.60±0.11	2.23±0.78	13.22 (4.16) e	3.85	8.25 (2.6) e
*S. uberis*	387	6.62	2.24	0.61±0.01	0.66±0.04	1.59	1.21	2.61
*S. zooepidemicus*	228	14.00	7.79	2.47±0.05	2.51±0.14	8.33	4.49	3.37
*E. faecalis*	261	12.35	2.73	0.73±0.05	0.65±0.03	7.53 (1.71) f	0.99	10.31 (2.34) f
*S. pyogenes*	209	10.11	3.92	0.75±0.02	0.75±0.05	2.01	1.44	2.68
*E. coli*	197	19.24	10.57	2.82±0.05	2.95±0.13	6.35	4.25	2.25
*S. suis*	170	25.35	13.50	3.23±0.11	2.85±0.32	9.24	7.17	2.86
*L. monocytogenes*	161	18.43	10.21	3.61±0.21	3.53±0.33	11.53	7.39	3.19
*Acb* complex (pubMLST.org)	137	20.98	10.98	1.94±0.09	1.87±0.35	10.92	7.11	5.62
*Acb* complex (Pasteur.fr)	84	19.89	17.57	3.21±0.56	2.76±1.01	11.49	11.49	3.57
*S. thermophilus*	84	5.03	3.77	0.57±0.03	0.64±0.08	1.83	1.83	3.21
*V. vulnificus*	81	10.79	7.76	2.32±0.04	2.33±0.35	3.96	3.85	1.70
*S. oralis*	77	27.94	22.51	6.46±0.26	6.40±0.44	11.18	10.44	1.73
*C. difficile*	50	5.04	4.61	0.64±0.15	0.72±0.21	3.31	3.27	5.17
*S. agalactiae*	46	2.23	1.85	0.50±0.02	0.49±0.03	1.04	0.98	2.08
*F. psychrophilum*	33	1.79	1.64	0.53±0.03	0.55±0.04	0.91	0.91	1.71
*L. casei* (Pasteur.fr)	32	1.99	1.96	0.39±0.02	0.40±0.02	0.68	0.68	1.74

a : Number of different STs in the population studied.

b : Standard deviation (SD) values from datasets containing more than 1500 STs were not calculated due to the limitations of the DnaSP software.

c : New π_MAX_ value and π_MAX_/π ratio after removal of 7 STs are indicated in parentheses.

d : New π_MAX_ value and π_MAX_/π ratio after removal of 17 STs are indicated in parentheses.

e : New π_MAX_ value and π_MAX_/π ratio after removal of 22 STs are indicated in parentheses.

f : New π_MAX_ value and π_MAX_/π ratio after removal of ST80 are indicated in parentheses.

As expected [44], the percentage of variable sites was found to be very sensitive to the sample size and it rapidly reached large values, close to site saturation and without biological meaning, as the sample size increased (*n*>500). This behavior illustrates how the use of this statistic to estimate DNA polymorphism in MLST studies may be misleading. By contrast, the nucleotide diversity (π) was only slightly affected by the sample size. However, it was found strongly affected by the set of loci selected, as illustrated by the significant (*p*<0.0001, Welch's test) differences between π values found when comparing the two MLST schemes developed for the *Acinetobacter calcoaceticus* - A. *baumannii* (*Acb*) complex [45], [46]. This indicates that π is inappropriate for comparing genetic diversity between bacterial species, unless using the same MLST scheme. In addition, π displayed high standard deviation values for some species, especially when the sample size was small (see for instance the values computed for 25 STs in *Enterococcus faecium* or *Acb* complex, Table 1). This statistic gives a global characterization of gene diversity and does not reveal sampling biases, such as errors in datasets (e.g. chimeric sequences or taxonomically misclassified isolates) or non-uniform population structures (e.g. the existence of independent genetic lineages within a species). These sampling biases were easily revealed by the maximal nucleotide diversity (π_MAX_), which is not directly sensitive to sampling size but only to the extreme values of sequence divergence, and by calculating the maximal to average pairwise nucleotide differences ratio (π_MAX_/π). For most species, this ratio was between 1.97 and 5.62, even for species known to contain several genetic lineages, for example *Listeria monocytogenes* [47]. In contrast, four species (*Streptococcus pneumoniae*, *Staphylococcus aureus*, *Enterococcus faecalis*, and *E. faecium*) displayed ratios of between 8.25 and 22.65, with π values generally higher than 10%. Phylogenetic trees computed with ST sequences from these species (data not shown) revealed that only few STs (less than 4% of the population) contributed to these high values, such that the values fell to become similar to those for other species after removal of these “outlier” STs. For instance, π_MAX_ value for *E. faecalis* dropped from 7.53% (π_MAX_/π ratio  = 10.31) to 1.71% (π_MAX_/π ratio  = 2.34) after removal of ST80, a chimeric ST made of *E. faecalis* and *E. faecium* sequences (data not shown). Similar chimeric STs also explained the aberrant values found for *E. faecium* (data not shown). This analysis led us to conclude that only π with its standard deviation, and π_MAX_ statistics were appropriate for estimating intraspecific genetic diversity of data samples and for detecting particular population structures or sample biases. In addition, it led us to assume that π_MAX_ might be suitable for comparing genetic diversity between bacterial species, even if estimated from different MLST schemes.

### Lactococcal strains involved in milk processing clustered in two clonal complexes

**Figure 1:**
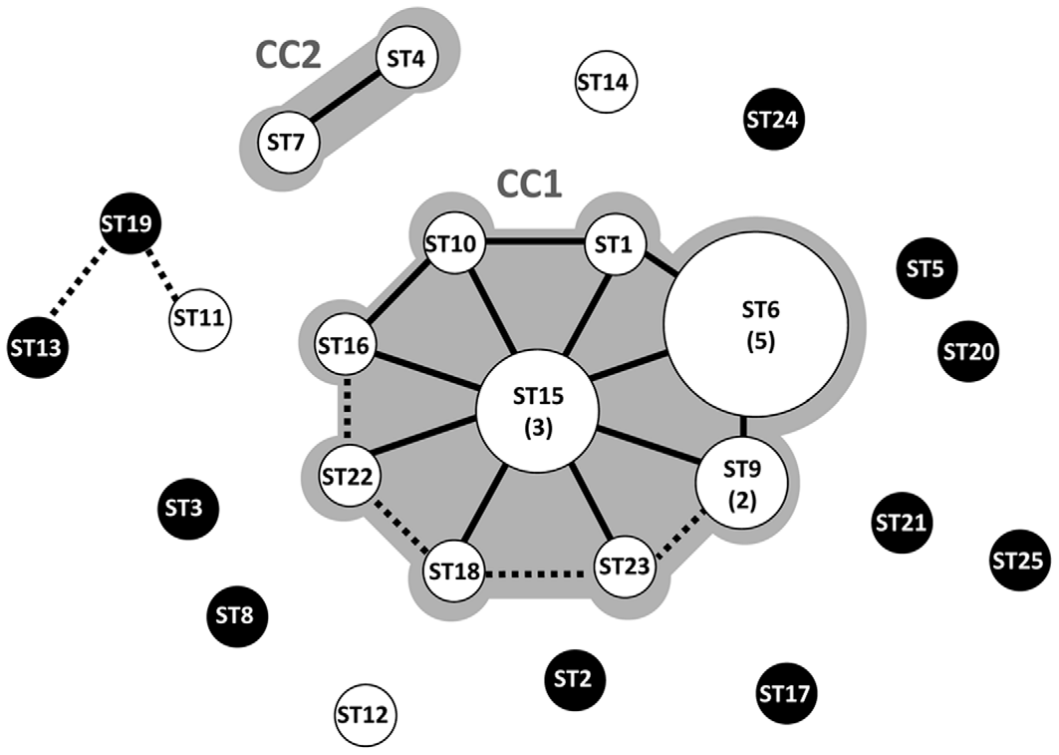
eBURST analysis of 32 *L. lactis* subsp. *lactis* strains. White circles correspond to dairy strains, and black circles to non-dairy strains. The size of the circles is proportional to the number of strains belonging to each ST (indicated in brackets). Clonal complexes (CC) are indicated in gray. Solid lines link SLV (Single Locus Variant, i.e. STs sharing five of the six loci). Dotted lines link DLV (Double Locus Variant, i.e. STs sharing four of the six loci). The ST15 is predicted to be the ancestor genotype of the major CC.

Twenty-two of the 25 STs included only single strains and the other three STs contained between two and seven strains (Table S1), with the most represented ST (ST6) including the reference strain IL1403 (with its parent IL594), LD01 (with its derivative, LD02), LD61 [48], LD90, and LD42. Genetic lineages in *L. lactis* subsp. *lactis* were identified by eBURST analysis [49], with clonal complex defined as group of STs sharing five of the six loci, and ancestor ST of each clonal complex defined as the ST with the highest number of neighboring STs (single locus variants, SLV). The 25 STs were distributed in 14 unique ST (singletons) and two clonal complexes (Fig. 1). The major clonal complex (CC1) included nine STs (corresponding to 20 strains) with ST15 identified as the ancestor genotype, whereas the second complex (CC2) comprised only two STs. A good correspondence was observed between strain origin and ST clustering, since the two clonal complexes (CC1 and CC2) contained only strains involved in milk processing (isolated from fermented products, or used as starters), with the exception of strains UCMA5713 and its [Lac]^-^ variant UCMA5733. A dairy origin was, however, strongly suspected for these two UCMA strains because both were isolated from grassland close to a dairy factory (N. Desmasure, personal communication); also, strain UCMA5713 rapidly ferments milk (data not shown) and contains both *lacE* and *prtP* genes (Table S1). In contrast, eleven of the 14 singletons corresponded to non-dairy strains. Relaxing the parameters for lineage definition to double-locus-variants (DLV, defined as STs sharing 4/6 loci) resulted in the merging of only three STs into a single group (ST11, ST13 and ST19, Fig. 1). The remaining 11 STs differ from each other by three to six loci, suggesting high level of genetic diversity among the corresponding isolates.

### Strains from the same ST reveal unexpected genome plasticity

**Figure 2:**
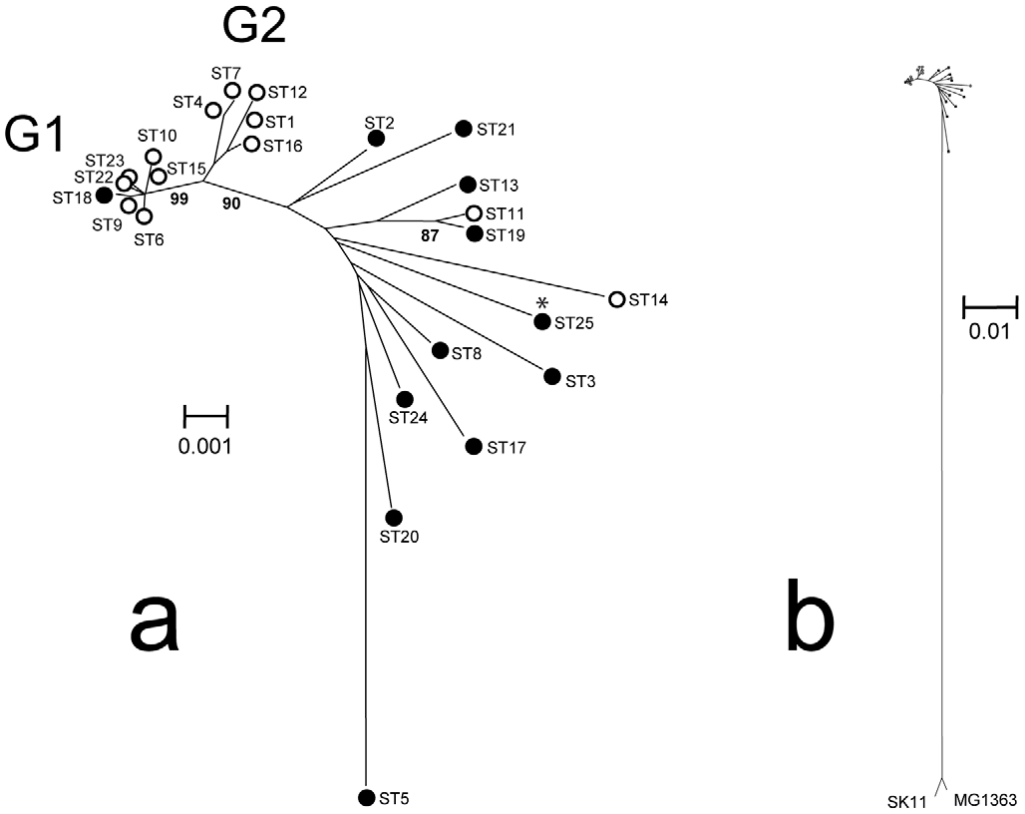
Phylogenetic relationships between lactococcal strains. The unrooted neighbor-joining tree (bootstrap 1000, Kimura 2-parameter model) was constructed from the 2,934-bp concatenated DNA sequences of the six loci. a) Tree constructed from the 32 subspecies *lactis* strains. b) Same tree after addition of the concatenated sequences from two subspecies *cremoris* strains. Only bootstrap values >80% are indicated. Open and closed circles correspond to dairy strains and non-dairy strains, respectively. The phylogenetic position of the recently sequenced strain KF147 [69] is indicated by an asterisk. This strain differs from strain NCDO2118 (ST25) by only one SNP.

The small contribution of homologous recombination to gene and genome evolution (see below) allowed strain relatedness to be assessed by classical tree-based phylogenetic analysis [42]. We used the neighbor-joining method [50] with concatenated sequences (2,934-bp) of the six loci (Fig. 2a). This analysis revealed that STs corresponding mainly to strains involved in milk processing (i.e. STs from CC1, CC2, and ST12) formed a genetic lineage distinct from other STs (bootstrap value 90%) and split in two clusters (G1 and G2, bootstrap value 99%). Except for the two strains isolated from animal skin (ST13 and ST19), which grouped together with one strain isolated from milk (ST11), the remaining STs corresponding to strains isolated from plants or raw milk showed no tendency to cluster. However, the genetic distance within the subspecies was far below the distance observed between *lactis* and *cremoris* subspecies, as assessed by including the corresponding 2,934-bp sequences of the two sequenced *cremoris* strains, MG1363 [21] and SK11 [51] (Fig. 2b). This strongly supports the notion that subspecies *lactis* and *cremoris* indeed constitute two distinct genetic lineages that presumably diverged a long time ago [21], [52].

**Figure 3:**
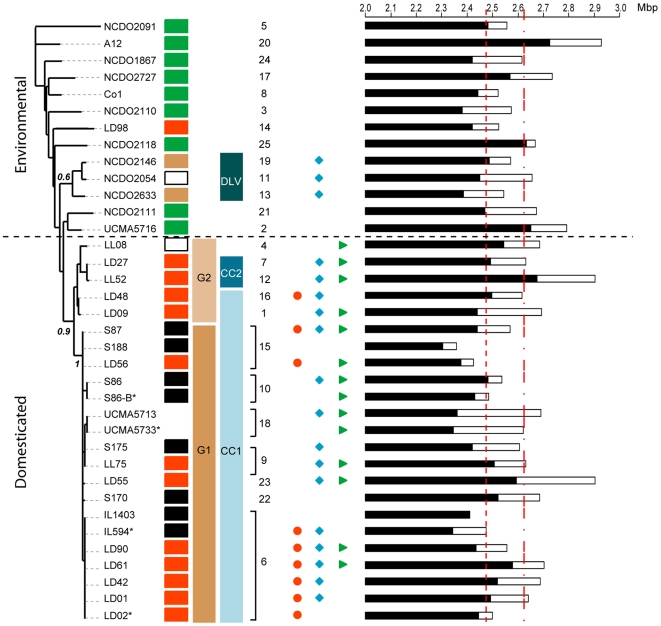
Comparison of gene-based phylogeny, strain origins, and genome properties. The genome features (chromosome/plasmid content size, presence of genes of industrial importance) of the 36 *L. lactis* subsp. *lactis* strains are compared to MLST-based strain relatedness. From left-to-right: neighbor-joining tree of the 36 strains, strain name; strain origin (color code: green  =  plant, brown  =  animal skin, white  =  milk, red  =  starter strains, black  =  cheese, unmarked  =  uncertain origin); genetic groups determined from the NJ tree; clonal complexes determined by eBURST; ST numbering; presence of *citP* gene (red dot), *lacE* gene (blue diamond), and *prtP* gene (green triangle); chromosome (black rectangles) and plasmid content (white rectangles) sizes. Derivative strains are indicated by an asterisk. The two dashed red lines indicate mean chromosome and genome size, respectively.

The genome relatedness of the 36 strains was estimated by computing Dice coefficients (S_D_) from pairwise comparisons of *Sma*I-macrorestriction patterns obtained by PFGE (Fig. S3a, provided as Supporting Information). Two-thirds of the strains (23/36) displayed values (S_D_<0.6) typical of unrelated strains [53], [54], with 55% of these values being between 0.11 and 0.35, the range observed when comparing the subspecies *cremoris* MG1363 strain to any subspecies *lactis* strain in the collection (yellow, Fig. S3a). This large diversity in genome fingerprints impeded robust UPGMA-based strain clustering, as no internal node was found to be significant when performing a bootstrap analysis (data not shown). Nevertheless, strains involved in milk processing were clearly separated from the other strains (compare Fig. 3 and Fig. S3b). However, strains belonging to the same ST were not necessarily clustered together, as fingerprints displayed unexpectedly low S_D_ values (0.44<S_D_<0.76 for strains belonging to ST6, S_D_<0.48 for strains belonging to ST15, and S_D_ = 0.4 for those from ST9, Fig. S3a). Since PFGE essentially monitors genome rearrangements rather than mutations, such S_D_ values strongly suggest high variability either in genome organization (for instance through rearrangements such as large inversions), or in genome content (through insertions/deletions of mobile genetic elements such as phages, ICEs, genomic islands etc.) within the subspecies *lactis*.

### *L. lactis* subsp. *lactis* is essentially clonal and displays low rate of recombination

We measured intergenic recombination by estimating the linkage disequilibrium between the six loci, using the standardized index of association statistic, *I*_A_^S^ [55]. To minimize linkage disequilibrium introduced by sampling bias or recent expansion of adaptive genotypes [56], only one sample from each ST was analyzed. A significant linkage disequilibrium was found when considering either the 25 STs of the collection (*I*_A_^S^ = 0.387, *p*<0.001) or the 14 singletons (*I*_A_^S^ = 0.1214, *p*<0.01), but not when grouping the STs from CC1 and CC2 (*I*_A_^S^ = 0.055, *p* = 0.198); this indicates that *L. lactis* subsp. *lactis* is essentially clonal. The intragenic recombination was estimated by empirical calculation of the per site ratio of recombination to mutation (*r/m*) statistic, which gives the relative probability that an individual nucleotide site will change by recombination or mutation [57]. Briefly, this method compares allelic variation from the ancestral ST to the SLV belonging to the clonal complex. If the variant allele differs by one SNP from the ancestral sequence, with this SNP not found in other ST, the nucleotide difference is counted as a point mutation (*m*), and if the variant allele either differs from the ancestral sequence by several SNPs, or is found in unrelated ST(s), these different nucleotides are considered originating from a recombination event (*r*). Three loci (*bcaT*, *pgk*, and *recN*) displayed allelic variation within the CC1 (Table S1), with eight allelic changes from the ancestor sequence (ST15) corresponding to 14 SNPs (Fig. S4). Four SNPs could be assigned to point mutations whereas ten SNPs were considered to have occurred by recombination, giving a per site *r/m* ratio of 2.5:1, a low value for a bacterial species [10], [36]. The main contributor for recombination events was *pgk.* Note that alleles 7 and 8 of this locus are closer to alleles 11 and 13 present in CC2 than to any allele present in CC1 (Fig. S4). Consequently, genetic variations at this locus may be responsible for the discrepancies in strain classifications observed between the allele- (eBURST, Fig. 1) and nucleotide-based (phylogenetic tree, Fig. 2a) methods. Both inter- and intragenic recombination tests, as well as the observation that only two loci (*tkt* and *metS*) displayed phylogenetic incompatibilities, among the 10 loci selected for the MLST scheme design, suggest that recombination may have happened, but has not played a major role in *L. lactis* subsp. *lactis* evolution.

### Gene diversity and evolution of lactococcal strains

We calculated the nucleotide diversity at each locus in *L. lactis* subsp. *lactis* (Table 2); it was from 0.66% for *bcaT* and *pepXP*, to 1.1% for *glyA*, with π_MAX_ ranging from 1.87% (*pgk*) to 3.07% (*recN*). These values confirmed the different evolution rates of the genes used in the new MLST scheme. In addition, the π values for *glyA* and *recN*, two loci whose variability is strongly correlated to the overall genome pair variability [37], were very close to the value obtained for concatenated sequences (see below). This validated the new MLST scheme as representative of the core genome relatedness within the subspecies *lactis*. We computed π and π_MAX_ from the concatenated sequences of the six loci: they were 0.82% (±0.1%) and 2.01%, respectively (Table 2). This π_MAX_ value falls within the range of values calculated for several species including *S. aureus* and some *Streptococci* (Table 1). However, the diversity was distributed unequally between strains of different origins, with strains involved in milk processing (cluster G1+G2) displaying almost fivefold lower diversity than other strains (Table 2). Thus, the non-dairy strains are the essential contributors to the genetic diversity within the subspecies. Inclusion into the analysis of DNA sequences from the subsp. *cremoris* strains MG1363 and SK11 not only raised the π and π_MAX_ values to 2.44% and 12.4% respectively, but also increased the π standard deviation to 0.98%, a value considered to be characteristic of highly divergent sequences (Table 1). These results strongly support the idea of the early separation of subspecies *lactis* and *cremoris* into two independent genetic lineages as suggested by the phylogenetic tree (Fig. 1b). Indeed, they indicate that the two subspecies should be analyzed separately in MLST studies.

**Table 2:** Gene diversity and evolution among *L. lactis* subsp. *lactis* strains.

Sequence	π (%)	π_MAX_ (%)	Tajima's *D*	Fu & Li's *D* a	Fu & Li's *F* a
Locus					
*bcaT* (516 bp)	0.66±0.15	2.51	−1.783 #	−2.116 #	−2.436 #
*glyA* (453 bp)	1.10±0.17	2.87	−0.672	−0.055	−0.281
*pdp* (492 bp)	0.82±0.14	2.85	−1.233	−2.125 #	−2.270 #
*pepXP* (504 bp)	0.67±0.16	2.58	−0.731	0.682	0.298
*pgk* (480 bp)	0.74±0.06	1.87	0.110	−0.736	−0.558
*recN* (489 bp)	0.95±0.15	3.07	−1.384	−1.367	−1.577
Concatenated sequence (6 loci, 2934 bp)					
25 STs	0.82±0.10	2.01	-	-	-
12 STs (cluster G1+G2)	0.23±0.03	0.40	-	-	-
13 STs (other)	0.99±0.12	2.01	-	-	-

# , 0.05<*p*<0.1 (two tailed test)

a , DNA sequences from the *cremoris* strain MG1363 were used as outgroup.

-, Not Determined.

A gene evolution model is generally established from MLST studies by computing the *dN*/*dS* ratio (ratio of the number of non-synonymous changes per non-synonymous site to the number of synonymous changes per synonymous sites). However, it has been demonstrated that the *dN*/*dS* ratio is not appropriate for inferring selection pressures from single bacterial populations, in which most differences between sequences represent segregating polymorphism rather than fixed substitutions, as assumed by the model [58]. Therefore, we developed a gene evolution model using the less controversial statistical tests of neutrality, such as the Tajima's *D* test [44], and the coalescent-based Fu & Li's *D* and *F* tests [59]. As substantial evidence indicates the separation of *cremoris* and *lactis* subspecies into two genetic lineages, the requirement of Fu & Li's *D* and *F* test for an outgroup sequence [59] could be fulfilled using DNA sequences from the subsp. *cremoris* strain MG1363. All three tests gave values that did not significantly deviate from zero (*p*>0.05, Table 2), indicating that the six loci evolved by random genetic drift.

### Genome properties are unrelated to strain origin or gene-based phylogeny

Although the first lactococcal genome sequence available was of a strain belonging to subspecies *lactis* [60], little is known about genome variability in this subspecies. In addition, previous analyses essentially focused on dairy strains [53], [61] and no information is available for strains of other origins. To estimate the extent of genome size differences between strains, and the contribution of plasmids and the chromosome to this genome size variation, various PFGE analyses were performed. Each strain contained from one to ten plasmids ranging from 2.2 kb to 120 kb in size (data not shown), making up 1% (35 kb, strain NCDO2118) to 12% (329 kb, strain UCMA5713) of the total genome (Table S1). Plasmid genetic markers determining metabolic properties important for dairy product manufacture, such as lactose (*lacE* gene) or casein catabolism (*prtP* gene), and citrate utilization (*citP* gene), were assigned to particular plasmids by Southern hybridization (Fig. 3, and Table S1). The two strains isolated from animal skin (NCDO2633 and NCDO2146) contained a copy of the *lacE* gene, but all other non-dairy strains had none of these markers. Among the dairy strains, nearly half (10/22) contained *prtP* and *lacE*, and the others either contained *prtP* (2/22) or *lacE* (6/22) alone, or neither (4/22). Therefore, although these two markers were generally associated with dairy strains, neither allowed unambiguous identification of strain origin. Strains containing the *citP* gene were grouped in three STs (ST6, ST15, and ST16) of the clonal complex CC1 (Fig. 3, and Table S1). This strongly supports the view that biovar *diacetylactis* may be distinguished by differences in chromosomal sequences not uniquely related to citrate utilization [62], and corresponds to a close genetic lineage among *L. lactis* subsp. *lactis* dairy strains.

**Figure 4:**
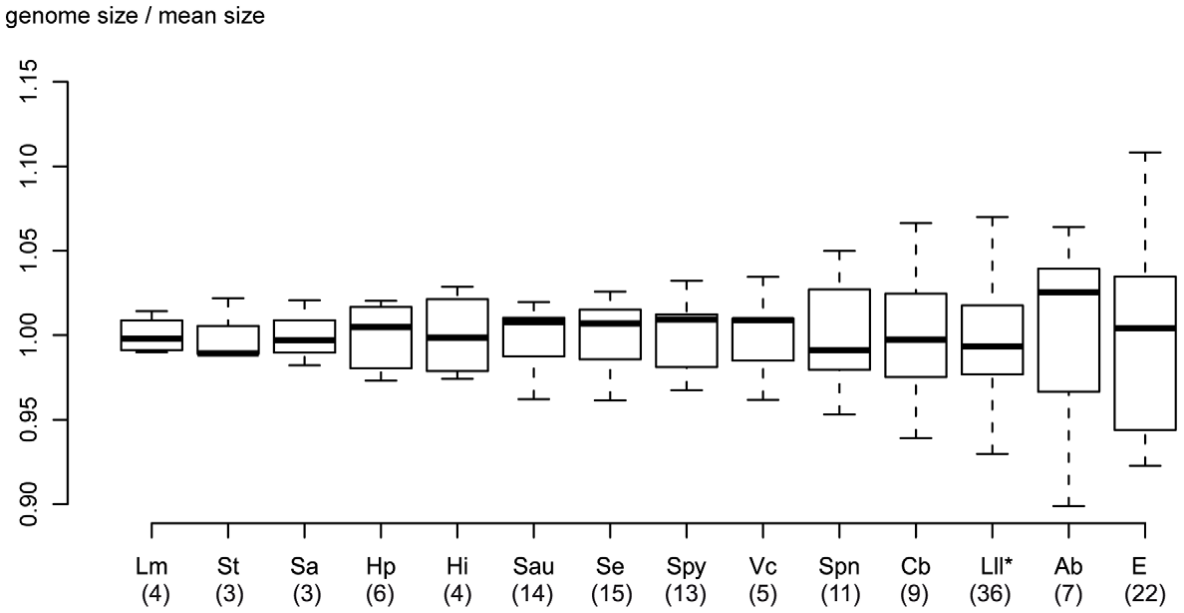
Comparison of chromosome size diversity within several bacterial species. For each species, the genome size distribution, summarized as a boxplot, is plotted according to the mean size. The species are ordered by increasing chromosome size diversity. Chromosomes sizes were obtained from sequence data (http://www.ncbi.nlm.nih.gov/genomes/genlist.cgi?taxid=2&type=1&name=Bacteria%20Complete%20Chromosomes). The number of chromosomes sequenced is indicated in brackets. Abbreviations: Lm, *L. monocytogenes*; St, *S. thermophilus*; Sa, *S. agalactiae*; Hp, *H. pylori*; Hi, *H. influenzae*; Sau, *S. aureus*; Se, *S. enterica*; Spy, *S. pyogenes*; Vc, *V. cholerae*; Spn, *S. pneumoniae*; Cb, *C. botulinum*; Lll, *L. lactis* subsp. *lactis*; Ab, *A. baumannii*; Ec, *E. coli*.

The mean chromosome size was 2475 kb overall with about 15% difference between the smallest (2,304±41 kb in strain S188) and the largest (2,725±72 kb in strain A12) (Fig. 3 and Table S1). This is a larger range than found for streptococcal species assumed to contain an “open” genome [63]. Indeed, this size spread ranks the subspecies *lactis* amongst bacterial species with high genome diversity (Fig. 4). The mean genome size for the different isolates (the sum of chromosome and all plasmids) was 2,619 kb, with about 20% difference between the extremes (2,359 kb in strain S188 and 2,930 kb in strain A12) (Fig. 3). Although the plasmid content significantly contributed to the genome size (Spearman ρ = 0.69, *p*<10^−5^), no correlation between chromosome and plasmid sizes was detected (Spearman ρ = 0.1, *p* = 0.57). In addition, no correlation was found between strain origin and the size of its plasmid content (Mann-Whitney test, *p* = 0.948), or its plasmid profile (data not shown). This was also true of other genomic characteristics (Fig. 3): there was no significant difference between dairy and non-dairy strains as concerns mean chromosome size (Mann-Whitney's test, *p* = 0.616), or mean genome size (Mann-Whitney test, *p* = 0.766). Lastly, the genomic features did not correlate with the MLST-based phylogenetic relationships between strains: some strains belonging to the same ST differed by up to 230 kb in total genome size (see for instance strains IL594 and LD61, Fig. 3), whereas some unrelated strains had similar chromosome sizes and plasmid contents (see for instance strains Co1 and LD02, Fig. 3).

## Discussion

To contribute to the characterization of the natural variability of *L. lactis*, we report a comparative evaluation of the genetic and genomic diversity of a collection of 36 strains isolated from different ecological sources and geographical areas. The various analyses revealed unexpectedly high variability within the subspecies *lactis* at both gene and genome levels, and gave clues about its population structure and evolution. These findings were not entirely coherent with the traditional division into dairy (i.e. isolated from dairy substrates) and non-dairy (i.e. isolated from other sources) strains, but rather support a new classification based on ecological separation between several ecotypes [64] corresponding to “domesticated” and “environmental” strains.

At the gene level, MLST analysis revealed two clonal complexes (CC1 and CC2) and 14 singletons. This genetic structure clearly clustered strains involved in milk processing, a human activity, and isolated from dairy starters (13 strains, Table S1) or fermented product (3 strains). These “domesticated” strains were almost exclusively found in the two clonal complexes, whereas “environmental” strains, isolated from various sources such as plant and animals (including raw milk), were scattered into unique STs. This demarcation was also observed in the phylogenetic tree built using the concatenated sequences, with “domesticated” strains clustering as a single clade that could be further decomposed into two genetic groups, G1 and G2 (with G1 including most strains from the biovar *diacetylactis*). In contrast, “environmental” strains were spread evenly across the phylogenetic tree and constitute the major contributors to the genetic diversity observed within the subspecies. The allelic distribution of all loci used in the MLST scheme strongly supported this opposition between the two ecotypes. This type of evolutionary pattern appears to be a general trend among the subspecies *lactis* and is not due to geographic sampling bias, because a similar separation has been observed when examining the phylogenetic tree produced by the alternative MLST scheme for lactococcal strains of other geographical origins [31].

The phylogenetic trees from both studies, rooted with strains from the subspecies *cremoris*, indicate that “environmental” strains appeared first, and that “domesticated” strains emerged only recently from a single founder event. It is assumed that high genetic diversity of “environmental” strains explains their ubiquitous presence in various natural environments (plants, animals and milk), but allows only poor growth during milk processing where they become a subdominant population. Such strains are expected to be only infrequently isolated from fermented products by standard bacteriological methods, as in the case of strains LD98 (Table S1 and Fig. 3) or ATCC 19435T [31]. This hypothesis is further supported by the identification of numerous “environmental” strains in raw milks from different areas (data not shown). The emergence of the “domesticated” strains through a single founder event suggests acquisition of adaptative mutations that allowed the descendant of this lineage to become the dominant *lactis* subspecies population during milk processing. Possibly, the founder event was acquisition of the plasmid-encoded genes involved in casein or lactose catabolism, because both genes i) are highly prevalent in the “domesticated” strains, ii) are undoubtedly advantageous for rapid growth in milk as strains containing both functions are able to ferment milk, and iii) the lactococcal plasmids are known to carry other functions of adaptive value in milk [65], [66]. However, in view of the versatility of such extra-chromosomal elements [67], [68], illustrated in this study by the complex distribution pattern of *prtP* and *lacE* genes with nearly half “domesticated” strains lacking one or both (Fig. 3), it appears more likely that there were several independent events, involving plasmid acquisition and loss. Indeed, the instability suggests that genes brought by these plasmids are not the key features responsible for the emergence of “domesticated” strains, and that only their presence in a subsample of the bacterial complex is essential, an assumption supported by the fact that artisanal whey, sourdoughs, and even defined commercial starters, are generally composed of several *L. lactis* strains.

In the absence of reliable universal molecular clock in bacteria [39], it is difficult to infer divergence times for the different evolutionary steps. Nevertheless, empirical cheese production at local scale by spontaneous fermentation or back-slopping over thousands of years would presumably have allowed the emergence of several independent genotypes adapted to milk processing; consequently, the uniqueness of the origin of “domesticated” strains, and low DNA polymorphism, are inconsistent with early lactococcal domestication of the order of 10,000 years ago. A simpler explanation would be that “domesticated” strains originate from a bottleneck event caused by the sampling of a very limited number of strains isolated from natural starters in the early 20^th^ century, when defined commercial starters were first used for standardized cheese production [15]. Subsequently, the emergence of the genetic group G1 is presumably associated with a second bottleneck allowing the emergence of fast acid-producing strains (corresponding to “modern” industrial strains) more adapted to the large-scale cheese production developed 40–50 years ago. These successive founder effects associated with human subsampling are supported by the different tests of neutrality, all of which indicate that each locus of the MLST scheme evolved by random genetic drift.

In contrast to the gene phylogeny, the macrorestriction analysis by PFGE did not allow robust strain clustering, except for few “modern” industrial strains of biovar *diacetylactis* belonging to the major ST (including the sequenced strain IL1403). In addition, the low S_D_ values within this ST revealed unexpectedly high genome plasticity within the subspecies, with most macrorestriction fingerprints being as disparate within the subspecies as between subspecies *lactis* and *cremoris*. As 84% of the *Sma*I restriction sites found in the KF147 chromosome [69] are also present in the IL1403 chromosome (data not shown), the low S_D_ values corresponded mostly to genome rearrangements such as inversions and insertion/excision of mobile genetic elements. This genome variability was also evident in the substantial variation in chromosome and total genome sizes (15% and 20%, respectively), indicating high fluctuation in strain-to-strain coding capacity. This range of genome size variability indicates that the pan-genome is as large as generally observed for species inhabiting diverse ecological niches, such as *Lactobacillus sakei* [70], *Pseudomonas aeruginosa* [71], and *E. coli* [72]. As most of the strains analyzed (28/36) have a chromosome larger than the IL1403 chromosome, this strain cannot be considered as representative of the coding capacities of the subspecies. In addition, genome characteristics (chromosome size, plasmid content size, plasmid profile, and total genome size) did not correlate with strain origin or with MLST-based phylogeny, with strains indistinguishable by MLST displaying up to 230 kb differences in genome size. This suggests that clonal diversification and phenotypic variability of the “domesticated” strains are largely the consequences of substantial genomic flux within the dispensable genome. Although large differences between the sizes of genomes of closely related strains has been suggested to be common in prokaryotes [73], this has been reported to date for only few proteobacteria, notably *Vibrio splendidus* [74], *Sinorhizobium meliloti* [75] and *E. coli* [5].

In conclusion, the core genome-based phylogeny substantiates early separation of the *L. lactis* subspecies *lactis* and *cremoris*, leads to the proposal of a new strain classification within the subspecies *lactis*, and suggests that there have been several genetic bottlenecks in the evolutionary history of strains involved in milk processing. The use of MLST will be of great help in defining the ecological and phylogenetic status of new lactococcal strains, and may be more informative than other genotyping methods. The high genome variability suggests a large pan-genome for the subspecies. However, this variability correlated with neither the strain origin nor the gene-based phylogeny, so numerous strains from the different ecotypes will need to be sequenced to characterize the lactococcal pan-genome.

## Materials and Methods

### Bacterial strains and culture conditions

*Lactococcus lactis* strains were obtained from various laboratory and industrial collections (LMGM-Toulouse, France for Sx strains; LMA-Caen, France for UCMAx strains; LBAE-Auch, France for the A12 strain; SOREDAB-La Boissiere Ecole, France for LLx and LDx strains). NCDO strains were obtained from the collection held at INRA (Jouy-en-Josas, France). Bacteria were grown at 30°C on M17-broth (Merck KGaA, Darmstadt, Germany) supplemented with 5 g.l^−1^ (w/v) of lactose or glucose. The lactose fermentation test was performed on milk-citrate BCP agar medium [76]. Strains are listed in Table S1 (provided as Supporting Information).

### DNA manipulation

Genomic DNA was extracted using the “DNeasy™ tissue” kit according to the manufacturer's instructions (Qiagen, Hilden, Germany). DNA probes corresponding to genetic markers of important industrial traits (*lacE*, encoding the lactose-specific Enzyme II of the PTS system; *prtP*, encoding the cell envelope-associated serine proteinase; and *citP*, encoding the membrane bound citrate permease involved in citrate uptake) were obtained by PCR amplification, and radiolabeled with dATP-^32^P using the “Megaprime™ DNA labeling system” (GE Healthcare Europe, GmbH). Restriction enzymes were purchased from New England Biolabs (Ipswich, USA). The automated RiboPrinter® (DuPont Qualicon, Wilmington, USA) device was used for *EcoR*I-ribotyping, according to the manufacturer's instructions. The V1-V4 region of the 16S DNA was amplified and double-strand sequenced (Eurofins MWG operon, Ebersberg, Germany), using primers E8_F (5′-AGAGTTTGATCCTGGCTCAG-3′) and E807_R (5′-TGGACTACCAGGGTATCTAATC-3′). Internal fragments of each of the six loci, *pepXP* (X-prolyl-dipeptidyl aminopeptidase), *recN* (ATPase involved in DNA repair), *pdp* (pyrimidine-nucleoside phosphorylase), *pgk* (phosphoglycerate kinase), *glyA* (serine hydroxymethyltransferase), and *bcaT* (branched-chain-amino-acid aminotransferase), were amplified and double-strand sequenced (Eurofins MWG operon, Ebersberg, Germany) using the primers listed in Table S2 (Supporting Information). Primers were designed by standard procedures using Clone Manager version 9.0 software (Sci-Ed Software). PCR conditions were: initial denaturation at 94°C for 3 min; 30 cycles at 94°C for 45 s, 55°C for 1 min, 72°C for 1 min using a MJ Mini thermocycler (Bio-Rad, Hercules, USA) in a 50 µl-mixture containing 10 ng of genomic DNA, 200 µM of each dNTP, 0.2 µM of each primer, 2.5 U *Taq* polymerase in 1x thermopol buffer (New England Biolabs). PCR products were purified using the “QIAquick PCR” Purification Kit (Qiagen). The quality of every sequence chromatogram was checked manually and each SNP was considered as correct if present on both DNA strands.

### PFGE analyses

Preparation of lactococcal DNA embedded in agarose matrix, digestion of DNA, pulsed field gel electrophoresis (PFGE), and Southern-blot with dried agarose gels were performed as previously described [77]. The size of each digested restriction fragment was estimated manually by comparison with either λ DNA concatemers (lambda ladder PFG marker, New England Biolabs) or *L. lactis* IL1403 *SmaI* restriction fragments [78], with PFGE conditions optimized for optimal resolution (pulse times of 2 to 210 s, depending on the fragment size to be determined). The size of the chromosome in each strain was estimated by averaging the sum of restriction fragment sizes calculated from either single *Sma*I digestion or double I-*Ceu*I/*Not*I digestions. *Sma*I endonuclease has previously been used to estimate the genome size of various lactococcal strains [53], [61] but generally cuts large plasmids in one or two fragments, leading to a slight overestimation of the chromosome size. In contrast, I-*Ceu*I and *Not*I do not cut lactococcal plasmids but generate a very large chromosomal fragment (>1.5 Mb) whose size is difficult to determine accurately by electrophoresis [79]. When applied to the IL1403 chromosome, this averaging method gave a value (2,411±14 kb) close to the size (2,365 kb) calculated from the chromosome sequence [60]. Plasmid DNA was linearized by S1 nuclease digestion [80]. Briefly, DNA embedded in agarose matrix was incubated at 37°C for 40 min with 2.5 units of S1 nuclease in 200 µl of 1x S1 buffer (Promega, Madison, USA). The reaction was stopped by adding 1 ml of TE 10/50 (Tris-Cl pH 8, 10 mM; EDTA 50 mM) and samples were kept on ice until PFGE electrophoresis.

### Computational analyses

The genomic relatedness of bacterial strains was estimated from pairwise comparisons of PFGE *Sma*I-macrorestriction patterns, and a matrix of binary data was constructed based on the presence/absence of each band. Dice coefficients (S_D_) for each pairwise comparison and corresponding genomic distances (1-S_D_) were calculated from the matrix using the WINBOOT program [81]. A UPGMA dendrogram was constructed with the NEIGHBOR program in the PHYLIP package v3.69 [82]. Bootstrap analysis of the UPGMA tree was performed using the WINBOOT program with 1000 pseudoreplications. For MLST analysis, forward and reverse DNA sequences were trimmed, aligned, and analyzed using MEGA4 v4.1 [83]. Conflicting phylogenetic signals were analyzed by split decomposition using SplitsTree v4.10 [42]. Allele and isolate dataset creation, arbitrary allele numbering, and Sequence Type (ST) assignation were done using mlstdbNet software [84]. STs clustering into clonal complexes (CC) and founder assignation were performed using eBURST [49]. Neighbor-joining trees (bootstrap 1000 using the Kimura two-parameter model [85]) were established with MEGA4 v4.1. The number of segregating sites (*S*), nucleotide diversity (π), Tajima's *D*, and Fu & Li's *D* and *F* values were calculated using DnaSP v5.10 [86]. The standardized index of association (*I*_A_^S^) was calculated using LIAN 3.5 (http://gump.auburn.edu/cgi-bin/lian/lian.cgi.pl). The MLST data from several bacterial species were downloaded from different MLST web sites (http://www.pasteur.fr/recherche/genopole/PF8/mlst/, http://www.mlst.net/, http://pubmlst.org/). Sequences with missing data were removed from the database by manual inspection using MEGA4 v4.1, and redundant sequences were removed using the NRDB program (http://pubmlst.org/perl/mlstanalyse/mlstanalyse.pl?site=pubmlst&page=nrdb&referer=pubmlst.org). π_MAX_ values were extracted from the squared similarity matrix calculated with the DNADIST program (D option set to “similarity table”) in the PHYLIP v3.69 package [82].

### Nucleotide sequence accession number

Allele sequences of the six MLST loci were deposited in Genbank/EMBL under the accession numbers HM597775 to HM597845. Sequence data are also available through our MLST web site (http://www-mlst.biotoul.fr/).

## Supporting Information

Table S1 *L. lactis* subsp *lactis* characteristics of the strains used in this study.
(PDF)

Table S2 Primers used for the MLST.
(PDF)

Figure S1 Split decomposition analysis of the different alleles at each individual locus. The conflicting phylogenetic tree topologies are illustrated by interconnected network. Numbers indicate allele number.
(PDF)

Figure S2 Location of loci used in the MLST scheme on the chromosome of IL1403 strain.
(PDF)

Figure S3 a) Matrix of S_D_ values for all pairwise comparisons of PFGE fingerprints. b) UPGMA dendrogram derived from the S_D_ values.
(PDF)

Figure S4 Polymorphic nucleotide sites found in the 32 *L. lactis* subsp. *lactis* strains at the six MLST genes. Only polymorphic sites are shown, with numbering starting at the beginning of the aligned sequence portion of each gene.
(PDF)
