# Supplementary material for: Genes but Not Genomes Reveal Bacterial Domestication of Lactococcus Lactis
Source: PLoS One. 2010 Dec 17;5(12):e15306. doi: 10.1371/journal.pone.0015306 (PMC3003715; doi:10.1371/journal.pone.0015306)
Supplement: Table S1 — L. lactis subsp lactis characteristics of the strains used in this study. (PDF) [file pone.0015306.s001.pdf]

Table S1. *L. lactis* subsp *lactis* characteristics of the strains used in this study.

Name [a]	Origin	ST	MLST allele number						Chromosome size [b]	Plasmid size	Southern-hybridization			*Eco*RI Ribotype	Reference
			bcaT	*glyA*	*pdp*	*pepXP*	*pgk*	*recN*			*citP*	*lacE*	*prtP*		
IL1403	laboratory strain, derivative of IL594	6	1	1	1	1	1	1	2411 ± 14	0	-	-	-	58-S-6	(Chopin et al. 1984)
IL594 (S491)	starter (Poligny, France)	(6) [c]	(1)	(1)	(1)	(1)	(1)	(1)	2344 ± 23	131	9	50		58-S-6	(Chopin et al. 1984)
LD90 (S461)	goat cheese (France), 2005	6	1	1	1	1	1	1	2437 ± 16	120	9	46	65	58-S-6	This study
LD01 (S451)	soft cheese starter (France), 1995	6	1	1	1	1	1	1	2493 ± 18	150	9	95	-	58-S-6	This study
LD02 (S453)	Brindza, goat cheese (Slovakia), 2004	(6) [d]	(1)	(1)	(1)	(1)	(1)	(1)	2445 ± 30	55	9	-	-	58-S-6	This study
LD42 (S465)	soft cheese starter (France), 1996	6	1	1	1	1	1	1	2521 ± 29	169	9	63	-	58-S-6	This study
LD61 (S460)	soft cheese starter (France), 2000	6	1	1	1	1	1	1	2579 ± 59	125	9	50	55	58-S-6	(Raynaud et al. 2005)
S87 (S479)	starter (France)	15	1	1	1	1	5	1	2442 ± 41	129	9	65	62	58-S-6	This study
LD56 (S467)	soft cheese starter (France), 2006	15	1	1	1	1	5	1	2377 ± 68	50	9	-	50	197-S-3	This study
S188 (S484)	starter (France)	15	1	1	1	1	5	1	2304 ± 41	55	-	-	-	1797-S-1	This study
LD48 (S457)	soft cheese starter (France), 1995	16	1	1	1	1	7	1	2498 ± 1 [b]	119	9	60	-	58-S-1	This study
LL75 (S468)	paneer cheese (Gurgaon, India), 2004	9	1	1	1	1	5	5	2509 ± 22	123	-	50	50	3766-S-7	This study
S175 (S482)	starter (France)	9	1	1	1	1	5	5	2420 ± 35	188	-	65	-	1797-S-1	This study
S86 (S477)	starter (France)	10	1	1	1	1	12	1	2482 ± 35	110	-	55	55	1797-S-1	This study
S86-B (S478)	[Lac]$^{-}$ derivative of S86	(10) [e]	(1)	(1)	(1)	(1)	(12)	(1)	2430 ± 18	55	-	-	55	1797-S-1	This study
UCMA5713 (S432) [f]	grassland (Normandy , France), 2003	18	1	1	1	1	5	11	2362 ± 14	329	-	65	92	1797-S-1	This study

UCMA5733 (S436) [f]	grassland (Normandy , France), 2003	(18) [g]	(1)	(1)	(1)	(1)	(5)	(11)	2348 ± 28	264	-	-	92	1797-S-1	This study
S170 (S480)	starter (France)	22	3	1	1	1	5	1	2522 ± 49	165	-	-	-	1797-S-1	This study
LD09 (S454)	Brindza, goat cheese (Slovakia), 2004	1	1	1	1	1	8	1	2442 ± 99	253	-	48	65	3338-S-4	This study
LD55 (S459)	Camembert (Normandy, France), 2006	23	10	1	1	1	5	1	2595 ± 93	309	-	75	120	1797-S-4	This study
LD27 (S508)	soft cheese starter (France), 1999	4	1	1	6	8	13	1	2492 ± 42	139	-	75	64	199-S-7	This study
LL52 (S466)	soft cheese starter (France), 2003	7	1	1	6	8	11	1	2677 ± 27	227	-	65	62	199-S-7	This study
LL08 (S464)	raw milk (Normandy , France), 2001	12	1	1	11	8	7	12	2545 ± 33	141	-	-	70	199-S-7	This study
NCDO2054 (S493)	milk, Slow lactose fermentation, 1976	11	2	2	2	2	3	3	2452 ± 27	205	-	74	-	nd [h]	
NCDO2146 (S497)	mastitis, 1979	19	2	11	13	2	3	3	2488 ± 21	84	-	84	-	199-S-1	
NCDO2633 (S495)	rectum of cow 330, 1981	13	2	2	2	2	2	2	2387 ± 21	159	-	90	-	nd [h]	
LD98 (S462)	paneer cheese (Gurgaon, India), 2004	14	4	4	3	4	6	6	2421 ± 40	105	-	-	-	520-S-2	This study
Co1 (S424)	corn (USA), 1993	8	2	3	7	7	3	3	2443 ± 69	80	-	-	-	199-S-1	(Salama et al. 1993)
NCDO2727 (S449)	mung bean (China), 1983	17	6	3	5	5	3	7	2571 ± 98	166	-	-	-	199-S-1	
NCDO2118 (S423)	frozen peas, 1978	25	9	9	10	10	10	10	2634 ± 11	35	-	-	-	3872-S-1	
UCMA5716 (S433)	grassland (Normandy, France), 2003	2	1	6	6	6	3	8	2651 ± 108	143	-	-	-	3872-S-4	This study
NCDO2111 (S450)	frozen peas, 1978	21	5	5	4	1	9	3	2471 ± 17	203	-	-	-	3872-S-5	
A12 (S473)	sourdough bread (Auch, France)	20	2	10	12	11	3	13	2725 ± 72	205	-	-	-	520-S-2	This study
NCDO2110 (S422)	frozen peas, 1978	3	7	7	8	2	13	3	2381 ± 31	195	-	-	-	3883-S-3	
NCDO2091 (S421)	seeds of Chinese radish (Japan)	5	8	8	9	9	10	9	2483 ± 18	75	-	-	-	3883-S-3	
NCDO1867 (S492)	frozen peas (England), 1966	24	2	3	2	3	4	4	2422 ± 68	194	-	-	-	3931-S-6	

[a]: alternative nomenclature

[b]: error calculated as the average deviation.

[c]: not determined (deduced from ST profile of its plasmid free derivative IL1403)

[d]: not determined (deduced from ST profile of strain LD01)

[e]: not determined (deduced from ST profile of strain S86)

[f]: these two closely related strains display archetypal phenotype and genotype of domesticated dairy strains.

[g]: not determined (deduced from ST profile of strain UCMA5713)

[h]: not determined (strains were not available at the time of analysis)

References

Chopin A, Chopin MC, Moillo-Batt A, Langella P. 1984. Two plasmid-determined restriction and modification systems in *Streptococcus lactis*. *Plasmid*. 11: 260-263.

Raynaud S, Perrin R, Cocaign-Bousquet M, Loubiere P. 2005. Metabolic and transcriptomic adaptation of *Lactococcus lactis* subsp. *lactis* biovar *diacetylactis* in response to autoacidification and temperature downshift in skim milk. *Appl Environ Microbiol*. 71: 8016-8023.

Salama MS, Sandine WE, Giovannoni SJ. 1993. Isolation of *Lactococcus lactis* subsp. *cremoris* from nature by colony hybridization with rRNA probes. *Appl Environ Microbiol*. 59: 3941-3945.
