# Supplementary material for: Genes but Not Genomes Reveal Bacterial Domestication of Lactococcus Lactis
Source: PLoS One. 2010 Dec 17;5(12):e15306. doi: 10.1371/journal.pone.0015306 (PMC3003715; doi:10.1371/journal.pone.0015306)
Supplement: Table S2 — Primers used for the MLST. (PDF) [file pone.0015306.s002.pdf]

Table S2. Primers used for MLST

Locus	Name (position [a])		PCR amplification [b]			Internal sequencing [b]
bcaT	bcaT_PCR_F	(1,322,807)	5'-AATTTAGACTGGGAAAATTTAGG-3'	bcaT_SEQ_F	(1,322,787)	5'-AGGATTCAGCTATCGGAACTTAC-3'
	bcaT_PCR_R	(1,321,829)	5'- CAACATCACCAAACTGAATG-3'	bcaT_SEQ_R	(1,322,166)	5'- TTTAGTATGTGTGCTTGGGTCAA-3'
glyA	glyA_PCR_F	(590,943)	5'-ATGATTTTTGATAAAGAAGATTTTGA-3'	glyA_SEQ_F	(591,069)	5'-GTTATGGCAGCACAAGGTTC-3'
	glyA_PCR_R	(592,077)	5'-CTTCAACTTCTTTAAATCCTCT-3'	glyA_SEQ_R	(591,622)	5'-GTTGTTGTAACGACATCAGCATAAG-3'
pdp	pdp_PCR_F	(1,465,707)	5'-ATGGTTGATCTCATTCAAAAGAA-3'	pdp_SEQ_F	(1,465,542)	5'-TCACGATGGCAATGGTTCATTC-3'
	pdp_PCR_R	(1,464,497)	5'-TTCAGTAACTAATTCGTCAG-3'	pdp_SEQ_R	(1,464,955)	5'-TCATTTCATTTCGATTTCCGATAGC-3'
pepXP	pepXP_PCR_F	(2,135,739)	5'-GGCTTATGGTGCTGCTACTACT-3'	pepXP_SEQ_F	(2,136,084)	5'-CTGGAATGTTACACCCGAACA-3'
	pepXP_PCR_R	(2,136,963)	5'-CACACTTTCAATAGGAATAATGAG-3'	pepXP_SEQ_R	(2,136,696)	5'-AGGAAGTTCAACCAAATCATCTAACA-3'
pgk	pgk_PCR_F	(242,797)	5'-TGGCAAAATTGACTGTAAAAGA-3'	pgk_SEQ_F	(242,999)	5'-CAAAGCTGGTAAATCACTTGCAC-3'
	pgk_PCR_R	(243,988)	5'-TTTTCAGTCAAAGCTGCAAGTC-3'	pgk_SEQ_R	(243,606)	5'-TGTGGTCAAGTGGCAAAATCAA-3'
recN	recN_PCR_F	(883,409)	5'-TTGTCGAATCAATGGTCAAATGG -3'	recN_SEQ_F	(883,611)	5'-CGTCAACAGCTCAATTTACGAC-3'
	recN_PCR_R	(884,272)	5'-TCCATATAAAGCTCAGAAAGTTCG-3'	recN_SEQ_R	(884,221)	5'-GCAATTTTATGGCGCGCTTC-3'

[a]: position of the first 5' base of the primer on IL1403 chromosome (GenBank accession number: NC_002662)

[b]: corresponding primers specific for subsp. *cremoris* strains are available upon request to the authors.
