# Supplementary material for: Genes but Not Genomes Reveal Bacterial Domestication of Lactococcus Lactis
Source: PLoS One. 2010 Dec 17;5(12):e15306. doi: 10.1371/journal.pone.0015306 (PMC3003715; doi:10.1371/journal.pone.0015306)
Supplement: Figure S1 — Split decomposition analysis of the different alleles at each individual locus. The conflicting phylogenetic tree topologies are illustrated by interconnected network. Numbers indicate allele number. (PDF) [file pone.0015306.s003.pdf]

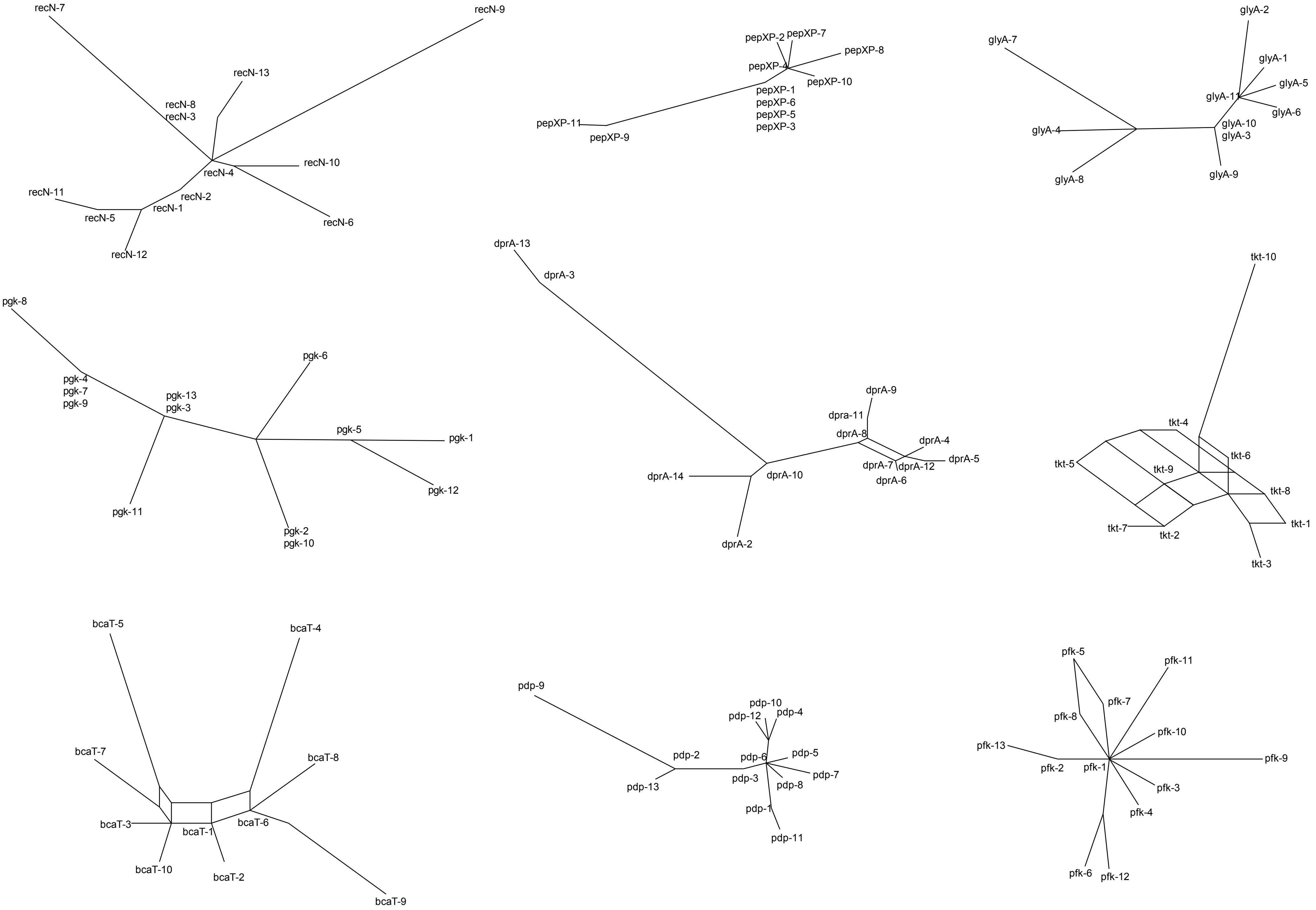

Fig. S1. Split decomposition analysis of the different alleles at each individual locus. The conflicting phylogenetic tree topologies are illustrated by interconnected network. Numbers indicate allele number
