# Supplementary figures and images for: Genes but Not Genomes Reveal Bacterial Domestication of Lactococcus Lactis
Source: PLoS One. 2010 Dec 17;5(12):e15306. doi: 10.1371/journal.pone.0015306 (PMC3003715; doi:10.1371/journal.pone.0015306)

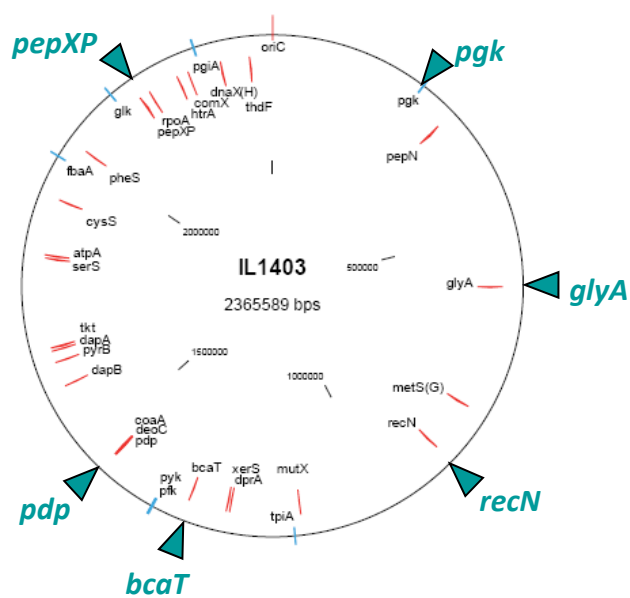

**Fig. S2.** Location of loci used in the MLST scheme on the chromosome of IL1403 strain.

Supplement: Figure S2 — Location of loci used in the MLST scheme on the chromosome of IL1403 strain. (PDF) [file pone.0015306.s004.pdf]
