# Supplementary material for: Genes but Not Genomes Reveal Bacterial Domestication of Lactococcus Lactis
Source: PLoS One. 2010 Dec 17;5(12):e15306. doi: 10.1371/journal.pone.0015306 (PMC3003715; doi:10.1371/journal.pone.0015306)
Supplement: Figure S3 — a) Matrix of SD values for all pairwise comparisons of PFGE fingerprints. b) UPGMA dendrogram derived from the SD values. (PDF) [file pone.0015306.s005.pdf]

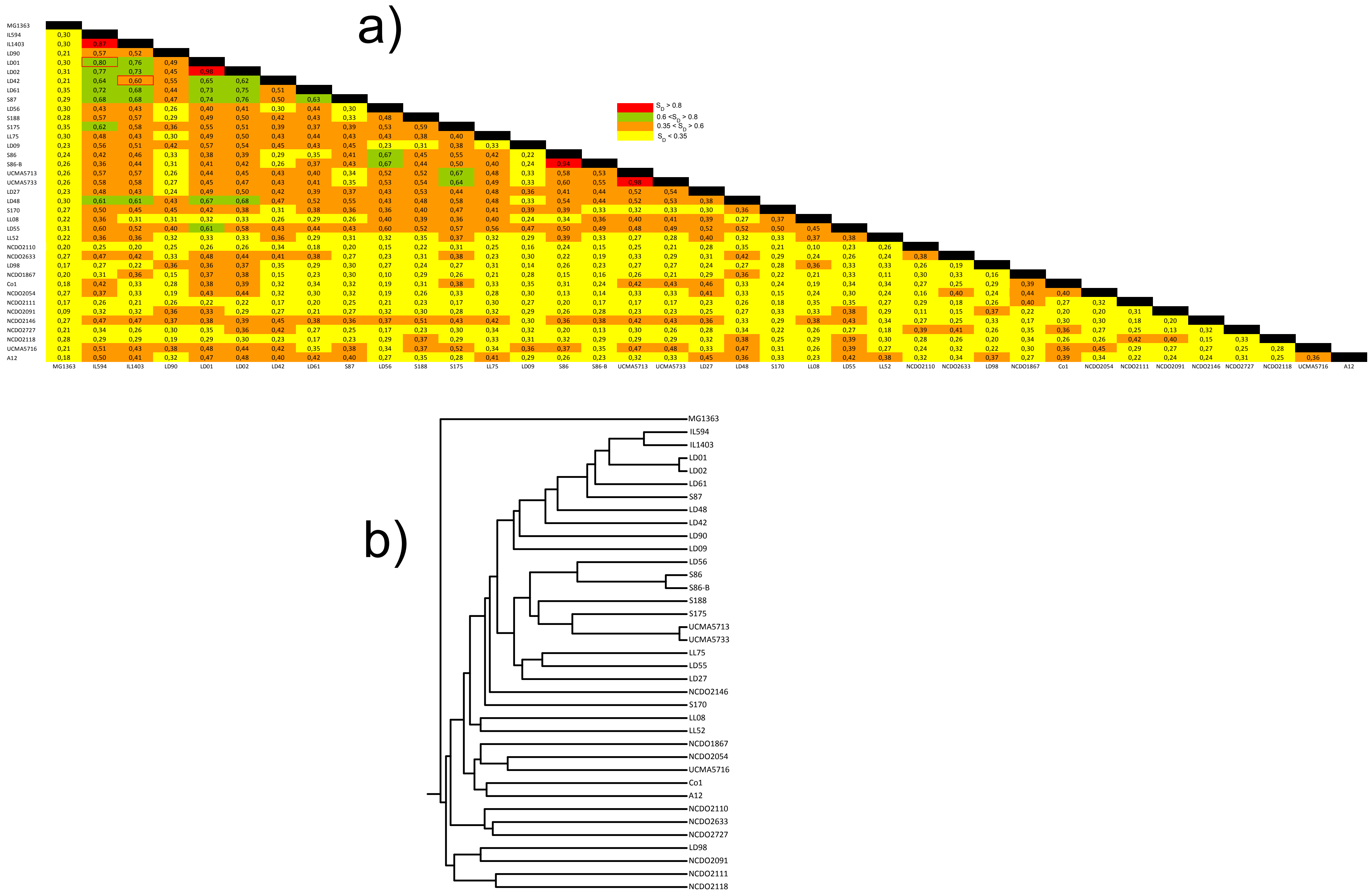

Fig. S3. a) Matrix of SD values for all pairwise comparisons of PFGE fingerprints. b) UPGMA dendrogram derived from the SD values.
