# Supplementary material for: Genes but Not Genomes Reveal Bacterial Domestication of Lactococcus Lactis
Source: PLoS One. 2010 Dec 17;5(12):e15306. doi: 10.1371/journal.pone.0015306 (PMC3003715; doi:10.1371/journal.pone.0015306)
Supplement: Figure S4 — Polymorphic nucleotide sites found in the 32 L. lactis subsp. lactis strains at the six MLST genes. Only polymorphic sites are shown, with numbering starting at the beginning of the aligned sequence portion of each gene. (PDF) [file pone.0015306.s006.pdf]

Fig. S4. Polymorphic nucleotide sites found in the 32 *L. lactis* subsp. *lactis* strains at the six MLST genes. Only polymorphic sites are shown, with numbering starting at the beginning of the aligned sequence portion of each gene.

	**4**	**8**	**48**	**72**	**105**	**113**	**165**	**170**	**175**	**240**	**264**	**267**	**315**	**316**	**324**	**327**	**354**	**369**	**438**	**441**	**453**	**456**	**480**	**510**
***bcaT*-1**	A	C	A	T	A	A	T	C	C	A	C	A	C	g	g	g	C	g	A	T	A	T	T	C
***bcaT*-2**	-	-	-	-	-	-	-	-	-	C	-	-	-	-	-	-	T	-	-	-	-	-	-	-
***bcaT*-3**	-	-	-	-	-	-	-	T	-	-	-	-	-	-	-	-	-	-	-	-	-	-	-	-
***bcaT*-4**	-	-	-	-	-	-	-	-	A	C	-	g	T	-	-	A	-	A	g	-	-	-	C	-
***bcaT*-5**	-	-	-	-	-	-	g	-	-	-	-	-	-	A	-	-	-	-	-	A	g	g	C	-
***bcaT*-6**	-	-	-	-	g	-	-	-	-	C	-	-	T	-	T	A	-	-	-	-	-	-	-	-
***bcaT*-7**	g	-	-	C	-	-	g	-	-	-	-	-	-	-	-	-	-	-	-	-	-	-	-	-
***bcaT*-8**	-	-	-	-	-	-	C	-	-	C	T	g	T	-	T	-	-	-	-	-	-	-	-	-
***bcaT*-9**	-	T	-	-	g	g	-	-	-	C	-	-	T	-	T	-	-	-	-	-	-	-	-	A
***bcaT*-10**	-	-	g	-	-	-	-	-	-	-	-	-	-	-	-	-	-	-	-	-	-	-	-	-

	**6**	**12**	**24**	**30**	**33**	**42**	**45**	**48**	**64**	**72**	**102**	**108**	**124**	**162**	**204**	**225**	**301**	**307**	**357**	**384**	**393**	**404**	**423**
***glyA*-1**	T	C	g	T	T	T	g	T	g	C	T	T	g	C	g	A	g	C	g	C	T	A	A
***glyA*-2**	-	-	-	-	-	-	-	-	-	-	-	-	T	-	-	-	A	g	-	-	-	-	T
***glyA*-3**	C	T	A	-	C	-	-	-	-	-	-	-	-	-	-	-	-	g	T	-	-	-	T
***glyA*-4**	-	T	A	C	-	-	-	-	A	-	-	-	-	-	-	g	-	g	-	T	C	-	T
***glyA*-5**	-	-	A	-	-	-	A	-	-	-	-	-	-	-	-	-	-	g	-	-	-	-	-
***glyA*-6**	-	-	-	-	C	C	-	-	-	-	-	-	-	-	A	-	-	g	-	-	-	-	-
***glyA*-7**	C	T	A	-	C	C	-	-	A	T	C	-	-	T	-	-	-	g	-	T	C	-	T
***glyA*-8**	C	T	A	-	C	-	-	g	-	-	-	g	-	-	-	-	-	g	-	T	C	-	T
***glyA*-9**	C	T	A	-	C	-	-	-	-	-	-	-	-	-	-	-	-	g	-	-	-	g	T
***glyA*-10**	C	T	A	-	C	-	-	-	-	-	-	-	-	-	-	-	-	g	-	-	-	-	T
***glyA*-11**	-	-	-	-	-	-	-	-	-	-	-	-	-	-	-	-	-	g	T	-	-	-	T

	**22**	**30**	**139**	**142**	**168**	**174**	**210**	**222**	**294**	**306**	**307**	**318**	**391**	**435**	**454**	**477**
***pepXP*-1**	T	T	C	g	A	T	g	C	T	T	g	A	C	g	C	g
***pepXP*-2**	C	-	-	-	-	-	-	T	-	-	-	-	-	-	A	-
***pepXP*-3**	-	-	-	-	-	-	-	T	-	-	-	-	-	A	-	-
***pepXP*-4**	C	-	-	-	-	-	-	-	-	C	-	-	-	A	-	-
***pepXP*-5**	C	-	-	-	-	-	-	T	-	-	-	-	-	-	-	-
***pepXP*-6**	-	-	-	-	-	-	-	T	-	-	-	-	-	-	-	-
***pepXP*-7**	C	-	-	A	-	-	-	T	-	-	-	-	-	-	-	-
***pepXP*-8**	-	-	T	-	-	-	-	-	-	-	-	T	-	-	-	-
***pepXP*-9**	C	C	-	-	T	C	A	T	C	C	-	-	T	A	-	-
***pepXP*-10**	-	-	-	-	-	-	-	-	-	-	-	-	-	-	-	T
***pepXP*-11**	C	C	-	-	T	C	A	T	C	C	T	-	T	A	-	-

	33	49	60	75	79	108	209	231	258	261	276	304	372	378	399	413	444	450	451	458	465	468	492
***pdp*-1**	C	A	g	T	g	g	g	C	A	C	T	C	T	A	A	g	T	C	A	g	C	C	g
***pdp*-2**	T	-	-	-	-	-	-	-	-	-	-	-	C	g	g	-	-	T	g	-	-	-	-
***pdp*-3**	T	-	-	-	-	-	-	-	-	-	-	-	-	-	-	-	-	T	g	-	-	-	-
***pdp*-4**	-	-	A	-	-	-	-	-	-	-	-	-	-	-	-	-	g	T	g	-	-	-	-
***pdp*-5**	-	-	-	-	A	-	-	-	-	-	-	-	-	-	-	-	-	T	g	-	-	-	-
***pdp*-6**	-	-	-	-	-	-	-	-	-	-	-	-	-	-	-	-	g	T	g	-	-	-	-
***pdp*-7**	-	-	-	C	-	A	-	-	-	-	-	-	-	-	-	-	-	T	g	-	-	-	-
***pdp*-8**	-	-	-	-	-	-	A	-	-	-	-	-	-	-	-	-	-	T	g	-	-	-	-
***pdp*-9**	T	-	-	-	-	-	-	T	g	T	C	T	C	g	g	-	-	T	g	-	g	-	A
***pdp*-10**	-	-	-	-	-	-	-	-	-	-	-	-	-	-	-	-	g	T	g	T	-	-	-
***pdp*-11**	-	-	-	-	-	-	-	-	-	-	-	-	-	-	-	-	-	-	-	-	-	T	-
***pdp*-12**	-	g	-	-	-	-	-	-	-	-	-	-	-	-	-	-	g	T	g	-	-	-	-
***pdp*-13**	T	-	-	-	-	-	-	-	-	-	-	-	C	g	g	A	-	T	g	-	-	-	-

	18	31	63	120	165	204	231	276	291	309	354	408	462
***pgk*-1**	C	g	C	C	C	T	C	T	A	T	C	g	T
***pgk*-2**	A	-	-	T	-	-	-	-	-	-	T	A	C
***pgk*-3**	A	-	-	T	-	C	T	-	-	-	-	-	-
***pgk*-4**	A	-	T	T	-	C	T	A	-	-	T	-	-
***pgk*-5**	A	-	-	-	-	-	-	-	-	-	-	-	-
***pgk*-6**	A	-	-	-	T	C	-	-	-	-	-	A	-
***pgk*-7**	A	-	-	T	-	C	T	A	-	-	T	A	-
***pgk*-8**	A	-	-	T	-	C	T	A	g	-	T	A	-
***pgk*-9**	A	-	-	T	-	C	T	A	-	-	T	-	-
***pgk*-10**	A	-	-	T	-	-	-	-	-	-	-	A	C
***pgk*-11**	A	A	-	T	-	C	T	-	-	-	T	A	-
***pgk*-12**	A	-	T	-	-	-	-	-	-	C	-	-	-
***pgk*-13**	A	-	-	T	-	C	T		-	-	T	A	-

	9	12	30	64	132	180	193	210	219	225	231	246	285	324	327	345	363	372	384	396	429	432	453	459	466	476	481
***recN*-1**	T	g	g	g	g	A	T	C	T	T	A	A	A	T	A	C	C	g	g	C	T	A	g	T	g	A	T
***recN*-2**	-	-	-	-	-	-	-	-	-	-	-	-	-	-	g	-	-	-	C	-	-	-	-	-	-	-	-
***recN*-3**	-	-	-	-	-	-	-	-	-	C	g	-	-	-	-	-	-	-	C	-	-	-	A	-	-	-	A
***recN*-4**	-	-	-	A	-	-	-	-	-	-	-	-	-	-	-	-	-	-	C	-	-	-	A	-	-	-	-
***recN*-5**	-	-	-	-	A	-	-	-	-	-	-	-	-	-	-	-	-	-	-	-	-	-	-	-	-	-	-
***recN*-6**	-	-	-	-	-	-	-	T	-	-	-	-	-	-	-	-	-	A	C	-	C	-	A	-	-	-	-
***recN*-7**	C	A	-	A	-	-	-	-	C		g	-	g	-	g	-	-	-	C	T	-	-	A	-	-	-	-
***recN*-8**	-	-	A	A	-	-	-	-	-	C	g	-	-	-	-	-	-	-	C	-	-	-	A	-	-	-	-
***recN*-9**	-	-	-	-	-	C	C		-	-	-	C	-	g	-	-	T	-	C	-	-	g	A	g	-	-	-
***recN*-10**	-	-	-	-	-	-	-	A	-	-	-	-	-	-	-	-	-	-	C	-	-	-	A	-	-	T	A
***recN*-11**	-	-	-	-	A	-	-	-	-	-	-	-	-	-	-	T	-	-	-	-	-	-	-	-	-	-	-
***recN*-12**	-	-	-	-	-	-	-	-	-	-	-	-	-	-	-	-	-	-	-	-	-	-	-	-	A	-	-
***recN*-13**	-	-	A	A	-	-	-	-	-	C	g	-	-	-	g	-	-	-	C	-	-	-	A	-	-	-	A
